# A Therapeutic Paradox: Intractable Hiccups in Acute Stroke and Vascular Parkinsonism Following Initiation of Carbidopa/Levodopa

**DOI:** 10.7759/cureus.109359

**Published:** 2026-05-21

**Authors:** Kevin Szafran, Aruba Asad, Naveed Tariq, Elliott J Berry, Ajendra Sohal

**Affiliations:** 1 Medicine, American University of the Caribbean School of Medicine, Florida, USA; 2 Physical Medicine and Rehabilitation, Nassau University Medical Center, East Meadow, USA

**Keywords:** acute inpatient rehabilitation, chlorpromazine, dopamine agonists, dopamine antagonists, dopamine receptors, intractable hiccups, levodopa-carbidopa, neurology case report, parkinsons disease, physical medicine and rehabilitation

## Abstract

Intractable hiccups are an uncommon but clinically significant condition that can lead to sleep disturbance, fatigue, impaired oral intake, and reduced quality of life. The hiccup reflex arc involves complex peripheral and central pathways, with dopaminergic signaling believed to play a modulatory role. In neurologically complex patients, distinguishing medication-related hiccups from structural central nervous system etiologies remains challenging.

We present a 94-year-old male with a history of vascular Parkinsonism and multiple comorbidities who was admitted for acute ischemic stroke involving the right frontal, parietal, and temporal lobes and insula. During his subsequent acute inpatient rehabilitation course, he developed new-onset intractable hiccups occurring approximately every 5 seconds and persisting during sleep, resulting in significant functional impairment. Initial management with metoclopramide and proton pump inhibitor therapy was ineffective, and no pulmonary or gastrointestinal etiology was identified. Given concern for a potential medication contribution in the setting of dopaminergic therapy initiated during hospitalization, carbidopa/levodopa was discontinued. Treatment with chlorpromazine led to progressive resolution of symptoms.

This case highlights a complex therapeutic and diagnostic dilemma in which acute cerebrovascular injury and dopaminergic therapy may have jointly contributed to the disruption of the hiccup reflex arc. It underscores the importance of considering both central neurologic and pharmacologic etiologies in intractable hiccups and illustrates the potential role of dopamine antagonism in symptom control within a medically and neurologically vulnerable population.

## Introduction

The management of intractable hiccups in patients with Parkinsonian syndromes presents a unique therapeutic and diagnostic challenge. While hiccups are typically benign and self-limited, persistent or intractable episodes can lead to significant morbidity, including sleep disturbance, fatigue, impaired oral intake, weight loss, and reduced quality of life, and may also serve as a clinical clue to underlying neurologic or systemic pathology [1].

The hiccup reflex arc involves complex peripheral and central pathways, including vagal and phrenic afferents, brainstem integration centers, and supratentorial modulatory regions. Disruption of this network at multiple levels may contribute to persistent hiccup generation. Acute ischemic stroke is a well-established cause of intractable hiccups, particularly when lesions involve the brainstem as well as supratentorial structures, such as the insular cortex and temporal lobe, which are increasingly recognized as important components of central autonomic and respiratory regulation [2]. In patients with acute cerebrovascular disease, distinguishing primary neurogenic hiccups from medication-associated etiologies may therefore be challenging, especially in the context of multimodal pharmacologic exposure affecting central neurotransmitter systems.

Dopaminergic agents, such as carbidopa/levodopa, are essential for the treatment of Parkinsonian motor symptoms but have been infrequently associated with the development of persistent hiccups [3]. Conversely, chlorpromazine, a dopamine D2 receptor antagonist with broader antihistaminergic, anticholinergic, and alpha-adrenergic properties, remains the only U.S. Food and Drug Administration-approved medication for intractable hiccups; however, its use may be limited in patients with underlying Parkinsonism due to the risk of worsening motor symptoms [4]. This opposing pharmacologic interplay further complicates management decisions in neurologically vulnerable patients.

We present a case of intractable hiccups occurring in the setting of acute ischemic stroke and vascular Parkinsonism following initiation of carbidopa/levodopa therapy, highlighting a complex therapeutic and diagnostic dilemma involving overlapping central neurologic injury and pharmacologic modulation of the hiccup reflex arc.

## Case presentation

Patient information

The patient is a 94-year-old male with a past medical history of prostate cancer, hypertension, hyperlipidemia, bilateral knee osteoarthritis, left eye blindness, osteomyelitis status post toe amputation, and vascular Parkinsonism. At baseline, prior to admission, he had mild asymmetric resting tremor, left greater than right, along with chronic cognitive impairment typically limited to orientation to person and place. He also had dysarthric speech, generalized weakness, and was wheelchair dependent for mobility. Neurologic examination at baseline demonstrated increased tone throughout, with evidence of Gegenhalten on the right and cogwheel or clasp-knife features on the left. Deep tendon reflexes were 2-3+ throughout. The patient had been diagnosed with vascular Parkinsonism approximately three years prior, but had not been followed longitudinally by neurology and was not receiving dopaminergic therapy. He presented after awakening with a left facial droop and inability to transfer from bed.

Physical examination

On admission, strength was 5/5 in the right upper and lower extremities. The left lower extremity demonstrated 4/5 strength, and the left upper extremity demonstrated 3/5 strength with an associated resting tremor. Deep tendon reflexes were normal bilaterally, and pathologic reflexes were absent. Sensation was intact throughout all extremities and the face. Muscle tone was normal without evidence of spasticity. Cranial nerve examination was notable for impaired visual acuity in the left eye; otherwise, cranial nerves were intact. Speech was dysarthric, and comprehension was preserved. The patient demonstrated poor static and dynamic balance in both sitting and standing positions.

Hospital course

Initial non-contrast CT head showed no acute intracranial pathology. MRI of the brain revealed multiple acute ischemic infarcts involving the right frontal, parietal, and temporal lobes, as well as the right insula. The patient was admitted to the medical intensive care unit for further management.

He was subsequently transferred to the general medical floor and then to the acute inpatient rehabilitation unit due to significant functional decline in mobility, balance, and activities of daily living. Given his history of Parkinsonism, carbidopa/levodopa 25 mg to 100 mg was initiated during hospitalization for symptomatic management.

During the rehabilitation course, the patient developed new-onset intractable hiccups occurring approximately every 5 seconds, including during sleep, resulting in significant disruption of sleep-wake cycles and oral intake. Initial treatment with metoclopramide 10 mg three times daily was ineffective. Pulmonology evaluation did not identify a respiratory etiology, and gastroenterology recommended proton pump inhibitor therapy for possible gastroesophageal reflux disease; however, symptoms persisted.

Chlorpromazine 10 mg every 12 hours was initiated while carbidopa/levodopa therapy was continued under neurology guidance. Due to persistent symptoms and concern for a possible medication contribution, carbidopa/levodopa was subsequently discontinued. Chlorpromazine dosing was escalated to 25 mg every 12 hours per neurology recommendations.

Given the known risk of severe motor deterioration and Parkinsonism-hyperpyrexia syndrome following abrupt withdrawal of dopaminergic therapy, the patient was closely monitored after discontinuation of carbidopa/levodopa. He demonstrated no acute worsening of rigidity, tremor, or autonomic instability. His functional limitations remained primarily attributable to multifocal ischemic stroke rather than progression of Parkinsonian motor symptoms.

Following these adjustments, the patient showed gradual improvement. Hiccups resolved during daytime hours and were no longer observed during therapy sessions, with only intermittent nocturnal episodes that decreased in frequency and severity. By discharge, he had significant improvement in sleep, oral intake, speech, and overall functional participation.

He was discharged in stable condition with chlorpromazine as needed and outpatient neurology follow-up for ongoing management of hiccups and Parkinsonism. A timeline of clinical events and interventions is summarized in Table 1.

**Table 1:** Clinical timeline and management of intractable hiccups MICU = Medical Intensive Care Unit; MRI = Magnetic Resonance Imaging; PT/OT = Physical Therapy/Occupational Therapy; FEES = Fiberoptic Endoscopic Evaluation of Swallowing; GI = Gastroenterology; PPI = Proton Pump Inhibitor; PO = By mouth (oral administration); mg = Milligrams; q = Every; q12h = Every 12 hours; TID = Three times daily; PRN = As needed

Hospital Day	Clinical Events	Intervention	Outcome
Day 1-2	Admission to MICU; MRI showed multifocal right hemispheric infarcts	Stroke workup	Stable
Day 3–4	Transfer to medicine floor	Continue medical management	Stable
Day 5	Transfer to acute inpatient rehabilitation	Initiation of PT/OT/Speech therapy	Functional deficits addressed
Day 6	FEES evaluation	Pureed diet, thin liquids, aspiration precautions	Safe swallowing established
Day 7	Stable rehabilitation course	Continue PT/OT/Speech therapy	Improvement in functional deficits
Day 8	Onset of intractable hiccups (q5 seconds, including during sleep)	Monitor hiccups	Sleep, speech, and feeding disruption
Day 9–11	Persistent hiccups	Metoclopramide 10 mg PO TID	No improvement
Day 11	Multidisciplinary evaluation	Pulmonology (no etiology); Gastroenterology (initiate PPI); Neurology → continue carbidopa/levodopa 25mg-100mg and start Chlorpromazine 10 mg PO q12h	No improvement
Day 12	Suspected medication-induced etiology	Carbidopa/Levodopa discontinued; Chlorpromazine 10mg continued	No improvement
Day 14	Ongoing symptoms	Chlorpromazine increased to 25 mg PO q12h	Notable reduction in frequency
Day 15–17	Gradual improvement	Continued chlorpromazine 25 mg PO q12h	Daytime hiccups resolved; intermittent nocturnal episodes only
Day 18 (Discharge)	Completed rehab	Discharged on PRN chlorpromazine; neurology follow-up planned	Significant improvement in sleep, speech, oral intake, and functional deficits

## Discussion

Intractable hiccups are an uncommon but clinically significant condition characterized by persistent involuntary contractions of the diaphragm and intercostal muscles. While often benign and self-limited, prolonged episodes can result in substantial morbidity, including sleep disturbance, fatigue, impaired oral intake, and reduced quality of life [5]. The hiccup reflex arc is complex, involving peripheral afferents (phrenic and vagus nerves), central integration within the brainstem, and efferent motor pathways. Emerging evidence suggests that central neurotransmitters, particularly dopamine, play a modulatory role in this reflex [6].

Medication-induced hiccups represent an underrecognized etiology, particularly in patients with underlying neurologic disease. However, an important limitation of this case is the presence of an acute multifocal ischemic stroke involving the right temporal lobe and insula, regions implicated in central modulation of the hiccup reflex arc. Central nervous system lesions, particularly involving the brainstem, as well as supratentorial structures, such as the insular cortex and temporal regions, are recognized causes of persistent and intractable hiccups. In this patient, the onset of hiccups occurred approximately eight days following acute stroke, making a cerebrovascular contribution a plausible competing etiology. Nevertheless, clinical features raised the consideration of a possible medication-related contribution. Symptoms developed during hospitalization after initiation of carbidopa/levodopa therapy and persisted despite initial therapies and multidisciplinary evaluation, with improvement following discontinuation of dopaminergic therapy and escalation of chlorpromazine. While this temporal relationship does not establish causality, it suggests that dopaminergic modulation may have contributed to hiccup generation in a neurologically vulnerable patient [7].

Management of intractable hiccups in patients with Parkinsonian syndromes presents a therapeutic challenge. Although abrupt withdrawal of dopaminergic therapy carries a known risk of Parkinsonism-hyperpyrexia syndrome, no such complications were observed in this closely monitored inpatient setting, likely reflecting relatively mild to moderate baseline vascular Parkinsonism and the predominance of acute stroke-related disability. Dopaminergic agents, such as carbidopa/levodopa, are central to motor symptom control but have been infrequently associated with hiccups. Conversely, dopamine antagonists are among the most effective pharmacologic treatments for hiccups. Chlorpromazine remains the only U.S. Food and Drug Administration-approved medication for this indication; however, its use is often limited in patients with Parkinsonism due to the risk of worsening motor symptoms, including rigidity and bradykinesia [8].

In this case, chlorpromazine was initiated at a low dose, with limited initial response, while dopaminergic therapy was continued. Following recognition of a possible medication contribution, carbidopa/levodopa was discontinued, and chlorpromazine was subsequently escalated, with gradual improvement in symptoms. The patient experienced resolution of daytime hiccups and progressive improvement of residual nocturnal symptoms, ultimately achieving significant symptomatic relief prior to discharge. Short-term use of chlorpromazine was well-tolerated, without clinically significant worsening of motor function.

Alternative agents for intractable hiccups include baclofen, gabapentin, and metoclopramide, though supporting evidence remains limited and responses are variable [9]. In this case, metoclopramide failed to provide symptomatic relief despite several days of therapy during concurrent dopaminergic treatment. This may reflect the multifactorial neurochemical basis of hiccups and the variable efficacy of single-agent dopamine receptor antagonism. Although both metoclopramide and chlorpromazine antagonize dopamine D2 receptors, chlorpromazine has broader receptor activity, including histaminergic, muscarinic, and alpha-adrenergic antagonism, which may contribute to more effective suppression of the hiccup reflex arc [10]. Importantly, chlorpromazine was initiated while carbidopa/levodopa was still being administered, with only a partial early response. Greater clinical improvement was observed following escalation of chlorpromazine dosing and subsequent dopaminergic withdrawal; however, the relative contributions of medication changes versus spontaneous neurologic recovery cannot be definitively determined.

This case highlights several important clinical considerations. First, medication-induced hiccups should be considered when a temporal relationship with pharmacologic therapy is present, particularly after exclusion of structural, infectious, and metabolic causes. Second, management of patients with Parkinsonian syndromes requires careful balancing of competing neurologic risks and therapeutic benefits. Finally, dopamine antagonists, such as chlorpromazine, may be effective even in medically complex patients when used cautiously and under close monitoring.

## Conclusions

Intractable hiccups in patients with Parkinsonian syndromes represent a rare but clinically challenging condition, particularly in the setting of acute neurologic disease. In this case, hiccups occurred in the context of acute ischemic stroke and vascular Parkinsonism following the initiation of dopaminergic therapy, suggesting a multifactorial disruption of the central hiccup reflex arc. Management requires careful consideration of competing neurologic risks, particularly when using dopamine antagonists, such as chlorpromazine, in patients with underlying Parkinsonism. Although the exact mechanism remains unclear, it likely reflects the combined effects of acute cerebrovascular injury and pharmacologic modulation of central dopaminergic pathways.
